# First Report of *Ignatzschineria indica* Infection in Greece and Review of the Literature

**DOI:** 10.3390/idr18040083

**Published:** 2026-08-07

**Authors:** Alexandra Vasilakopoulou, Evaggelia Oikonomoula, Vasiliki Anagnosti, Pavlos Siozos, Ioannis Liakopoulos, Eleni Kalogeropoulou, Sofia Damianidou, Polyxeni Karakosta, Sophia Vourli, Panagiota-Christina Georgiou, Anastasios Ioannidis, Spyros Pournaras

**Affiliations:** 1 Laboratory of Clinical Microbiology, Attikon University Hospital, Athens Medical School, National Kapodistrian University of Athens, 12462 Athens, Greece; leeoik@icloud.com (E.O.); vasoanagnosti@gmail.com (V.A.); ekaloger@yahoo.com (E.K.); sofia-dam@hotmail.com (S.D.); p_karakosta@hotmail.com (P.K.); pcgeorgiou@gmail.com (P.-C.G.); tasobi@gmail.com (A.I.); spournaras@med.uoa.gr (S.P.); 2 2nd Department of Internal Medicine—Propaedeutic, Attikon University Hospital, Athens Medical School, National Kapodistrian University of Athens, 12462 Athens, Greece; pavsioz@yahoo.gr (P.S.); giannisliako@hotmail.com (I.L.); 3 Laboratory of Bacteriology and Antimicrobial Resistance Mechanisms, Institute of Biosciences and Applications, National Centre of Scientific Research Demokritos, 15341 Athens, Greece; vourli@bio.demokritos.gr; 4 Department of Nursing, Faculty of Health Sciences, University of the Peloponnese, 22131 Tripoli, Greece

**Keywords:** *Ignatzschineria*, bacteraemia, myiasis

## Abstract

Background: *Ignatzschineria* species are rare emerging human pathogens that are typically associated with myiasis. Methods: We report the first case of *Ignatzschineria indica* bacteraemia in Greece in a male patient presenting with maggot-infested wounds of the thorax, shoulders, and arms. The isolate was identified by matrix-assisted laser desorption/ionization time-of-flight mass spectrometry (MALDI-TOF MS), and antimicrobial susceptibility testing was performed according to EUCAST guidance. A review of the published literature on *I. indica* bacteraemia was also conducted. Results: The patient was diagnosed with bacteraemia caused by a multisusceptible *I. indica* isolate and was successfully treated with antibiotic therapy, resulting in complete recovery. This represents the first reported case of *I. indica* infection in Greece. Review of the literature showed that most published *I. indica* isolates were susceptible to a broad range of antimicrobial agents, and all reported patients survived, although some required limb amputation because of severe underlying soft tissue disease. Conclusions: *Ignatzschineria indica* infection should be considered in patients with neglected, maggot-infested wounds. Increased awareness of this emerging pathogen, together with the use of modern microbiological identification methods, may improve recognition of these uncommon infections. Socio-economic inequalities and the changing distribution of insect vectors due to climate change may contribute to the occurrence of such infections.

## 1. Introduction

*Ignatzschineria* is a Gram-negative, aerobic, non-spore-forming, non-motile, rod-shaped bacterium that belongs to the class Gammaproteobacteria. This genus has been isolated from the first and second larval stages of the obligate parasitic fly *Wohlfahrtia magnifica* [1]. *W. magnifica* is mostly found in southern Europe, Central Asia, the Middle East, North Africa, and China [2,3,4]. The larvae of *W. magnifica* can infest the tissues of vertebrate animals and humans (myiasis) [5]. *W. magnifica* is an important veterinary pathogen causing traumatic myiasis primarily in sheep, but also in goats, cattle, horses, dogs and other mammals. Human myiasis represents an accidental infestation and is typically associated with neglected wounds, poor hygiene, or debilitating underlying conditions [6]. *Ignatzschineria* species are emerging pathogens that have been isolated from clinical samples from patients with myiasis [7]. The genus *Ignatzschineria* was first proposed in 2007 to replace the genus *Schineria* [8]. *Ignatzschineria indica* has been described as a cause of human wound infection after myiasis since 2014 [9]. The designation ‘emerging pathogen’ reflects the increasing number of published human cases over the past decade, largely due to improved detection by MALDI-TOF MS and molecular methods, together with reports from an expanding geographic distribution.

*Ignatzschineria indica* grows well on blood agar with visible non-haemolytic colonies after 24 h of aerobic incubation at 37 °C, while growth on MacConkey agar is slower and needs 48 h incubation (lactose-negative colonies). Catalase and oxidase reactions are positive [1]. Until now, there have been a handful of cases from Europe describing *Ignatzschineria* infection (France, Spain, The Netherlands, Denmark). Moreover, internationally there have been a few more reported cases of human *I. indica* infection from USA, Canada, Argentina, Japan [10,11].

## 2. Case Presentation

A 62-year-old male was brought to a tertiary university hospital in Athens, Greece by Emergency Medical Services (EMS). He had poor health status and was found lethargic with limited communication ability. He was living on the street in squalid conditions and had a history of mental illness and alcohol abuse. On physical examination he looked ill and had numerous open wounds on the anterior and posterior thoracic wall, on the shoulders and arms. The lesions were tender and erythematous and infested with numerous maggots. His body temperature was 36.8 °C. His blood pressure was 103/68 mm Hg, peripheral oxygen saturation 99%, and heart rate 99 beats/min. Lung auscultation revealed decreased breath sounds. The remaining physical examination was unremarkable. Hematological and biochemical investigations showed a white blood cell count of 7.1 × 10^9^ L^−1^ (reference range 4–10 × 10^9^ L^−1^) with 76.7% neutrophils, C-reactive protein 338 mg L^−1^ (reference range < 5 mg L^−1^) and overall normal results for hemoglobin level, platelet count, liver enzymes, electrolytes and creatinine. The abdominal ultrasound examination and head CT scan were normal. A chest X-ray revealed mild pulmonary infiltrates on both lungs. Two blood culture sets inoculated in aerobic and anaerobic bottles (BACTEC Plus Aerobic/F and Anaerobic/F Culture Vials, Becton Dickinson) were drawn upon admission and empirical treatment with ampicillin/sulbactam was initiated (3 gr × 4 IV). The wounds were irrigated with water and washed with chlorhexidine solution.

**Figure 1:**
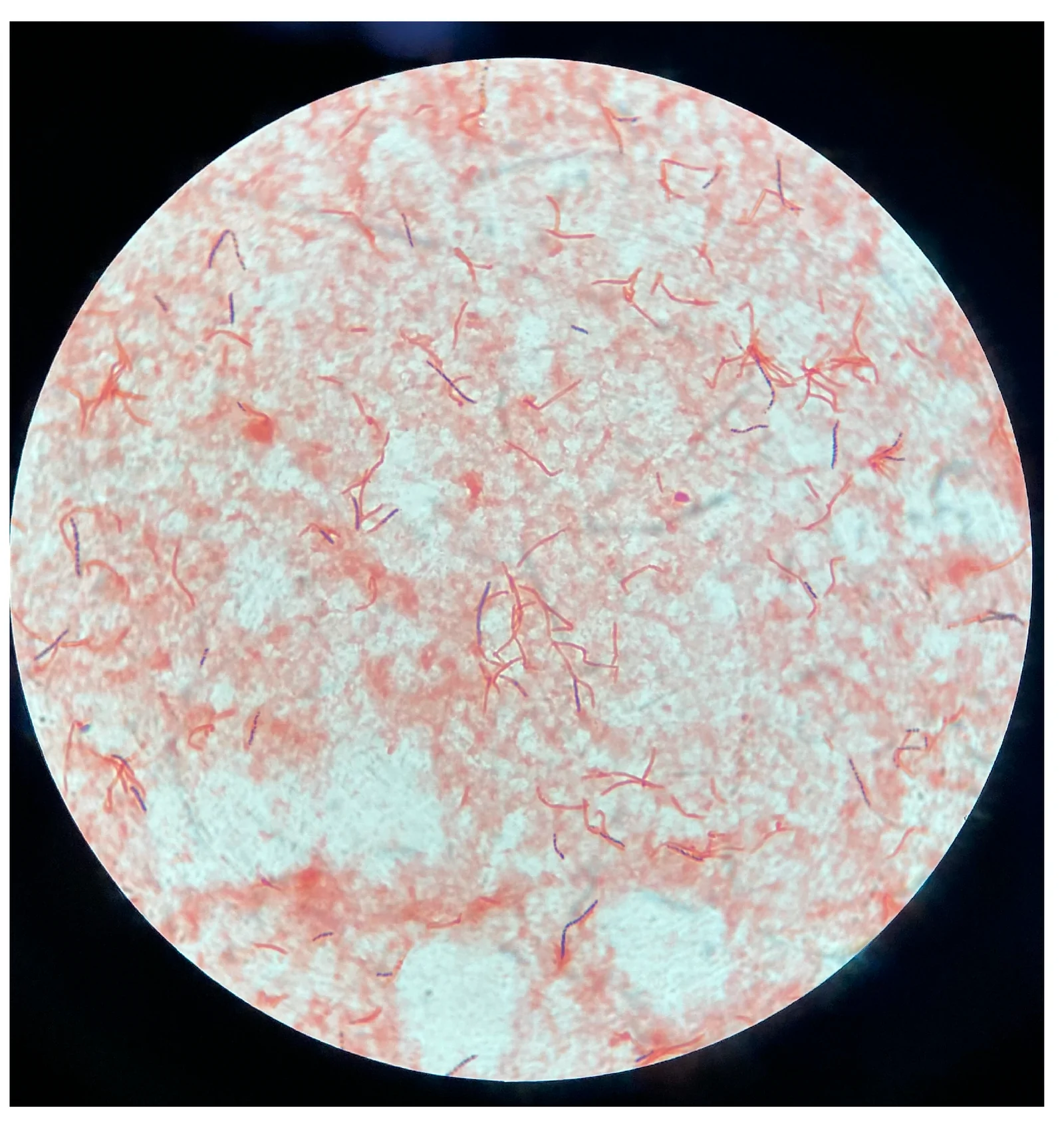
Gram stain of *Ignatzschineria indica* from blood culture. (Gram variable rods).

One of the aerobic bottles revealed growth after incubation for 48 h. Gram-variable bacilli were seen on microscopy (Figure 1). The blood culture broth was inoculated to blood agar, chocolate agar and MacConkey agar plates (Oxoid, Basingstoke, UK), and after aerobic incubation for 24 h small colonies were grown on blood and chocolate agar and after 48 h non-lactose-fermenting colonies were grown on MacConkey agar. Gram stain from the growth on solid media revealed Gram-negative rods. Basic biochemical identification testing showed an oxidase- and catalase-positive strain. Further workup was performed using matrix-assisted laser desorption/ionization time of flight mass spectrometry (MALDI-TOF MS, Microflex LT, Bruker Daltonics, Bremen, Germany) and interpretation of the result was done by MBT Compass Software (Version 4.1) and Spectra Library: MBT 8468. The MBT Compass Software matches an organism’s acquired mass spectrum against reference spectra and assigns an arbitrary log score value from 0.00 to 3.00 reflecting pattern similarity, the identification score criteria are: (a) ≥2.00 to 3.00: Secure/reliable identification at the species level; (b) ≥1.70 to 1.99: Reliable identification at the genus level only; (c) <1.70: Unreliable identification [12].

In this case the bacterium was identified as *Ignatzschineria indica* with a highly confident score of 2.52. Identification was also performed by the Vitek 2 GN card (BioMérieux, Marcy-l’Étoile, France), which resulted in a low confidence identification for *Sphingomonas paucimobilis* (VITEK 2 ID probability: 90%). For VITEK 2 bacterial identification at the species level is divided into four groups based upon the probability of accurate identification as follows: excellent (probability of accurate identification, ≥96%), very good (93 to 95%), good (89 to 92%), and acceptable (85 to 88%) [13]. The 90% ID probability is considered marginally good and it was finally disregarded because *Shingomonas paucimobilis* is a microorganism that does not grow in MacConkey agar [14]. Vitek 2 GN does not have the biochemical profile of *Ignatzschineria* in its library of identifiable microorganisms [15]. Susceptibility testing was performed using MIC test-strips (Liofilchem, Roseto degli Abruzzi, Italy) on Mueller–Hinton agar (Oxoid, Basingstoke, UK). There are no specific susceptibility breakpoints for this rare pathogen and thus the EUCAST guidance document ‘When-there-are-no-breakpoints’ was used to interpret the results [16]. All MICs were in the susceptible range, being for ampicillin 0.016, amoxycillin/clavulanic 0.016, ceftriaxone 0.002, aztreonam 0.064, piperacillin/tazobactam 0.125, meropenem 0.016 and ciprofloxacin 0.016 μg/mL.

The patient’s therapy was changed on the fourth day of hospitalization to piperacillin/tazobactam (4.5 gr × 4 IV) for a total of two weeks. Repeat blood cultures were all negative and the patient was discharged after 18 days of hospitalization. The hospital’s social services found a secure new home for the patient and scheduled regular psychiatric care. Upon a follow up visit, the wounds completely healed up.

## 3. Discussion–Review of the Literature

**Figure 2:**
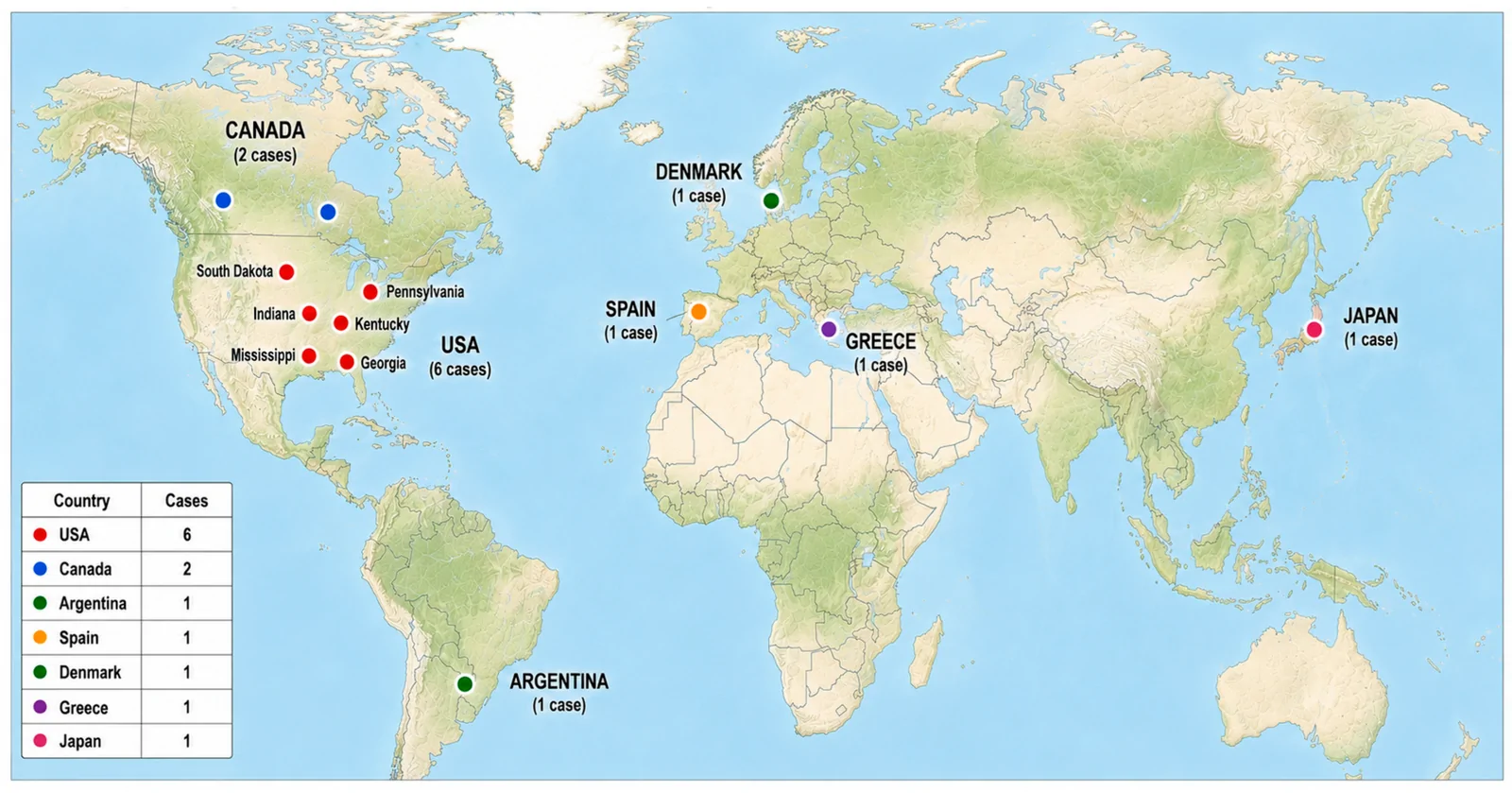
Geographic distribution of published *Ignatzschineria indica* Bacteraemia cases worldwide (n = 13).

Four *Ignatzschineria* species have been described until now, *I. indica*, *I. larvae*, *I. ureiclastica* and *I. cameli*. Flesh flies are the natural hosts for *Ignatzschineria* species and all human infections by this microbial genus have been reported to have an association with maggot infestation of wounds (myiasis) [17]. The first two cases of *Ignatzschineria* human infection were reported from France in 2007, and both patients suffered from myiasis and bacteraemia [18,19]. In the last 15 years more cases of myiasis associated *Ignatzschineria* bacteraemia have been recognized [7,9,11,20,21,22,23,24,25,26,27,28]. Additionally, a case of *Ignatzschineria* urinary tract infection has been described from USA [9]. In Table 1 there is a summary of all the published *I. indica* bacteraemia cases from around the world. After 2020 most of the cases have been identified by MALDI-TOF. Moreover the majority of *I. indica* isolates were susceptible to most antibiotics tested and all of the patients were cured. Some cases required amputation. In the published cases, amputations were performed because of advanced tissue necrosis, osteomyelitis, severe peripheral vascular disease or extensive chronic wounds, rather than because of *Ignatzschineria* infection itself. In Figure 2 the wide geographical distribution of *I. indica* bacteraemia cases is presented.

Infections by *Ignatzschineria* are probably underdiagnosed and thus underreported due to the unusual biochemical phenotype of the genus and the fact that these bacterial species are not included in the databases of the common commercial biochemical identification kits. In our laboratory, the semiautomated biochemical identification of the isolate by Vitek 2 (BioMerieux, Marcy-l’Étoile, France) showed *Sphingomonas paucimobilis* (with identification score of 90%). A MALDI–TOF mass spectrum for *Ignatzschineria indica* has been added to the Bruker reference library in 2016 [29]. We identified the bacterium by MALDI-TOF MS as *Ignatzschineria indica.* The presence of maggots in the patients’ wounds corroborated our diagnosis. Before the availability of MALDI-TOF MS identification, microbiology laboratories had to rely primarily on 16S rRNA gene and *gyrB* sequencing for *Ignatzschineria* accurate identification [9,18,19,20,21]. These molecular methods were performed usually in reference laboratories and consequently there was considerable delay in diagnosis.

*Ignatzschineria indica* also shares ecological and clinical characteristics with *Wohlfahrtiimonas chitiniclastica*, another fly-associated Gram-negative bacterium causing wound infections and bacteraemia in patients with myiasis. Although they belong to different genera, both are members of the class Gammaproteobacteria, inhabit necrophagous fly larvae, and have likely been historically underrecognized before the introduction of MALDI-TOF MS and molecular identification methods. The accumulating literature on both organisms suggests that fly-associated bacteria have to be considered in patients presenting with myiasis [26].

Although molecular confirmation by 16S rRNA gene sequencing, gyrB sequencing, or whole-genome sequencing was not performed for this case, we believe the identification was reliable. The MALDI-TOF MS score (2.52) exceeded the manufacturer’s threshold for secure species-level identification. In contrast, the alternative biochemical identification obtained by the VITEK 2 GN system was considered unreliable because *Ignatzschineria* spp. are not represented in its database and because the suggested organism, *Sphingomonas paucimobilis*, is not expected to grow on MacConkey agar; whereas, our isolate clearly did. We nevertheless acknowledge that sequencing-based confirmation would have further strengthened the microbiological characterization of this isolate and should be considered in future reports whenever feasible.

The case we describe presented with the wound form of myiasis as a complication of neglected wounds on the chest. Myiasis is usually reported from tropical climates but it can be seen also in other geographic regions especially in poor homeless people who cannot care adequately for their personal hygiene [30]. Our patient fitted this typical low socioeconomic status, suffered from mental illness and was homeless. Unfortunately, maggots had been rapidly discarded after cleaning the wounds, and we could not have an entomological identification. We assume that the larvae present in the patient’s wounds most probably belonged to *Wohlfahrtia magnifica*, since this species is known to occur in Southern Europe. However, reports from North and South America, where *W. magnifica* is absent, suggest that *Ignatzschineria* spp. may also be transmitted by other necrophagous flies, including species of the genus *Lucilia*. These observations indicate that the ecological association between *Ignatzschineria* and fly larvae is likely broader than originally recognized and may vary according to geographic region [31].

Fortunately, most of the *Ignatzschineria* strains that have been studied until now were susceptible to beta-lactams, aminoglycosides, fluoroquinolones, colistin, and trimethoprim/sulfamethoxazole [5,9,20,22,23]. There has been only one report of a carbapenem-resistant *Ignatzschineria* [10]. The *Ignatzschineria* strain that we isolated had low MICs for ciprofloxacin and all beta-lactams tested and our patient was treated with piperacillin/tazobactam showing a full recovery.

The clinical and laboratory findings in this report add information to the accumulating data on the emerging pathogen *Ignatzschineria* and its geographic distribution. This is the first case report of *I. indica* infection in Greece.

## 4. Conclusions

We should always consider how social and economic inequalities may lead to unusual infections. Moreover, global climate changes may contribute to a wider distribution of insect vectors. We have to keep in mind the possibility of *Ignatzschineria* infection in patients with neglected wounds that are infested with maggots.

Finally, whenever feasible, larvae recovered from myiasis-associated wounds should be preserved for entomological identification, as this may improve our understanding of the insect vectors involved and their associated microbiota. Although *Ignatzschineria indica* remains an uncommon cause of human infection, the increasing number of reported cases from different geographic regions, together with improved detection by MALDI-TOF MS and molecular techniques, supports its recognition as an emerging pathogen whose epidemiology and clinical spectrum are still being defined.

## Figures and Tables

**Table 1:** *Ignatzschineria indica* Bacteraemia published cases.

Country	Age/ Sex	Risk Factors	Primary Site	Identification Method	Antibiotic Susceptibility	Treatment	Outcome	Year/ Reference
USA Kentucky	64 M	Homeless, myiasis	Foot wound	16S rRNA	No data	SAM + VA → cefalexin	Toe amputation; survived	2014 [9]
USA South Dakota	67 M	Alcoholism, chronic ulcers, myiasis	Foot osteomyelitis	16S rRNA	Broad susceptibility (TZP: intermediate)	TZP + CM → CIP +VA	Below knee amputation; survived	2014 [9]
USA Mississippi	46 M	Chronic Kidney Disease, gout, decubitus ulcers, myiasis	Sacral ulcers, osteomyelitis	16S rRNA	Broad susceptibility	TZP → cefepime + MTZ → LVX	Cure	2017 [22]
USA Indiana	37 M	Myiasis, poor hygiene	Lower extremity ulcers	No data	No data	VA + CM + TZP → cefepime	Cure	2018 [5]
USA Pennsylvania	82 M	Peripheral vascular disease, chronic wounds, poor hygiene, myiasis	Lower extremity ulcer	MALDI-TOF MS	Multi-susceptible (TET: intermediate)	Cefepime → ceftriaxone	Cure	2020 [4]
Canada	60 M	Homelessness, myiasis	Necrotic leg wound	MALDI-TOF MS + 16S rRNA	Broad susceptibility	TZP + VA	Cure	2020 [25]
Canada	Old No data	Diabetes, poor living conditions, myiasis	Heel ulcer	MALDI-TOF MS + 16SrRNA	Susceptible to all	TZP	Cure	2020 [7]
Argentina	72 M	Homelessness, alcoholism, myiasis	Ulcer in the anterion area of left tibia	16S rRNA	CIP susceptible	CIP + CM	Amputation; survived	2018 [23]
Spain	44 M	Homelessness, alcoholism, poor hygiene, myiasis	Leg and foot ulcers	MALDI-TOF MS + 16SrRNA	Broad susceptibility	AMC	Cure	2017 [11]
Denmark	67 M	Diabetes, vascular disease, myiasis	Ulcer in amputated right foot	MALDI-TOF MS + Whole Genome Sequencing	Broad susceptibility	TZP + Cloxacillin → CIP	Survived (below knee amputation)	2026 [26]
Japan	70 sF	AdenoCa, poor hygiene, myiasis	Occipital lesion	MALDI-TOF MS	Broad susceptibility (SXT: resistant)	Ceftriaxone	Cure	2025 [27]
USA Georgia	72 M	Homelessness, IV drug use, recurrent cellulitis	Left foot wound	Outside Lab molecular identification	No data	TZP → LVX	Cure	2024 [28]
Greece	62 M	Homeless, mental illness, alcohol abuse, myiasis	Thoracic wall, shoulders and arms wounds	MALDI-TOF MS	Broad susceptibility	SAM → TZP	Cure	2026 This paper

Abbreviations: AMC: Amoxicillin-clavulanate; CIP: Ciprofloxacin; CM: Clindamycin; LVX: Levofloxacin; MTZ: Metronidazole; SAM: Ampicillin-Sulbactam; SXT: Trimethoprim-Sulfamethoxazole; TET: Tetracycline; TZP: Piperacillin-Tazobactam; VA: Vancomycin.

## Data Availability

The data generated and analyzed during this study are not publicly available due to patient privacy and ethical restrictions. De-identified data may be available from the corresponding author on reasonable request, subject to institutional review and approval.

## References

[1] Gupta A.K., Dharne M.S., Rangrez A.Y., Verma P., Ghate H.V., Rohde M., Patole M.S., Shouche Y.S. (2011). *Ignatzschineria indica* sp. nov. and *Ignatzschineria ureiclastica* sp. nov., isolated from adult flesh flies (Diptera: Sarcophagidae). Int. J. Syst. Evol. Microbiol..

[2] Martínez I.R., Leclercq M. Data on Distribution of Screwworm Fly *Wohlfahrtia magnifica* (Schiner) in Southwestern Europe (Diptera: Sarcophagidae). Notes Fauniques de Gembloux. https://www.cabdirect.org/cabdirect/abstract/19950503483.

[3] Tóth E.M., Hell É., Kovács G., Borsodi A.K., Márialigeti K. (2006). Bacteria isolated from the different developmental stages and larval organs of the obligate parasitic fly, *Wohlfahrtia magnifica* (Diptera: Sarcophagidae). Microb. Ecol..

[4] Snyder S., Singh P., Goldman J. (2020). Emerging pathogens: A case of *Wohlfahrtiimonas chitiniclastica* and *Ignatzschineria indica* bacteremia. IDCases.

[5] Lysaght T.B., Wooster M.E., Jenkins P.C., Koniaris L.G. (2018). Myiasis-induced sepsis: A rare case report of *Wohlfahrtiimonas chitiniclastica* and *Ignatzschineria indica* bacteremia in the continental United States. Medicine.

[6] Sotiraki S., Farkas R., Hall M.J.R. (2010). Fleshflies in the flesh: Epidemiology, population genetics and control of outbreaks of traumatic myiasis in the Mediterranean Basin. Vet. Parasitol..

[7] Deslandes V., Haney C., Bernard K., Desjardins M. (2020). *Ignatzschineria indica* bacteremia in a patient with a maggot-infested heel ulcer: A case report and literature review. Access Microbiol..

[8] Tóth E.M., Borsodi A.K., Euzéby J.P., Tindall B.J., Márialigeti K. (2007). Proposal to replace the illegitimate genus name *Schineria* Tóth et al. 2001 with the genus name *Ignatzschineria* gen. nov. and to replace the illegitimate combination *Schineria larvae* Tóth et al. 2001 with *Ignatschineria larvae* comb. nov. Int. J. Syst. Evol. Microbiol..

[9] Barker H.S., Snyder J.W., Hicks A.B., Yanoviak S.P., Southern P., Dhakal B.K., Ghimire G.R., Couturier M.R. (2014). First case reports of *Ignatzschineria* (*Schineria*) *indica* associated with myiasis. J. Clin. Microbiol..

[10] Maniam K., Argentine S. (2022). A case of sepsis due to a rare carbapenem-resistant *Ignatzschineria* species. IDCases.

[11] Rodríguez-Zúñiga D., González-Galiano N., Leal-Negrado Á., Hidalgo-Pérez E. (2019). First case of sepsis by *Ignatzschineria* in Spain associated with myiasis. Description of a case and review of the literature. Enferm. Infecc. Microbiol. Clín..

[12] Calderaro A., Chezzi C. (2024). MALDI-TOF MS: A Reliable Tool in the Real Life of the Clinical Microbiology Laboratory. Microorganisms.

[13] Zbinden A., Böttger E.C., Bosshard P.P., Zbinden R. (2007). Evaluation of the colorimetric VITEK 2 card for identification of gram-negative nonfermentative rods: Comparison to 16S rRNA gene sequencing. J. Clin. Microbiol..

[14] Gursoy S., Yasar K.K., Sari N.D., Kuvat N., Ozturk S. (2018). Sphingomonas Paucimobilis Bacteremia in a Hemodialysis Patient and Literature Review. Int. J. Crit. Care Emerg. Med..

[15] BioMerieux Webpage. https://www.biomerieux.com/.

[16] EUCAST Guidance Document on When There Are no Breakpoints. https://www.eucast.org/bacteria/clinical-breakpoints-and-interpretation/when-there-are-no-breakpoints/.

[17] Tsang C.C., Tang J.Y.M., Fong J.Y.H., Kinne J., Lee H.H., Joseph M., Jose S., Schuster R.K., Tang Y., Sivakumar S. (2018). *Ignatzschineria cameli* sp. nov., isolated from necrotic foot tissue of dromedaries (*Camelus dromedarius*) and associated maggots (*Wohlfahrtia* species) in Dubai. Int. J. Syst. Evol. Microbiol..

[18] Roudiere L., Jean-Pierre H., Comte C., Zorgniotti I., Marchandin H., Jumas-Bilak E. (2007). Isolation of Schineria sp. from a man. Emerg. Infect. Dis..

[19] Maurin M., Delbano J.N., Mackaya L., Colomb H., Guier C., Mandjee A., Recule C., Croize J. (2007). Human infection with *Schineria larvae*. Emerg. Infect. Dis..

[20] Cecile L.B., Martin G., Sylvie R., Emmanuelle M., Philippe L. (2015). Association of Necrotizing Wounds Colonized by Maggots with *Ignatzschineria*–Associated Septicemia. Emerg. Infect. Dis..

[21] Heddema E., Janssen F., van Westreenen H. (2016). A case of *Ignatzschineria bacteraemia* in an unconscious man from the Netherlands. JMM Case Rep..

[22] Muse H., Jenkins R.L., Oliver M.B., Kim S., Grantier R.L., Malhotra B.K., Parham J.J., Stover K.R. (2017). A Case of *Ignatzschineria indica* Bacteremia following Maggot Colonization. Case Rep. Infect. Dis..

[23] Cipolla L., Derdoy L., Archuby D., Tarzia A., Govedic F., Prieto M. (2018). Sepsis secondary to complicated skin and soft tissue infection caused by *Ignatzschineria indica*. First case report in Latin America. JMM Case Rep..

[24] Do S.R., Mitra S., Garces C.C., Anwar F. (2021). *Ignatzschineria* spp. bacteremia from maggot infestation. IDCases.

[25] Fear T., Richert Q., Levesque J., Walkty A., Keynan Y. (2020). *Ignatzschineria indica* bloodstream infection associated with maggot infestation of a wound in a patient from canada. J. Assoc. Med. Microbiol. Infect. Dis. Can..

[26] Christiansen R.J., Mortensen C., Cebula W., Laugesen A.L., Mortensen J.F., Kemp M., Engsbro A.L. (2026). The first report of *Ignatzschineria indica*, *Ignatzschineria ureiclastica* and *Wohlfahrtiimonas chitiniclastica* Bacteremia in two patients with fly larvae-infested wounds in Scandinavia. IJID Reg..

[27] Mura T., Takahara Y., Iguchi M., Ueda N., Iinuma Y. (2025). Polymicrobial bacteremia including *Ignatzschineria indica* caused by myiasis in a female patient with carcinoma of unknown primary. J. Infect. Chemother..

[28] Strike A., Alaws H., Welch B., Ignatowicz A. (2024). *Ignatzschineria indica* Bacteremia Initially Misdiagnosed in a Patient with a Maggot-Infested Wound. Cureus.

[29] Bruker Corporation (2016). MBT 6903 MSP Library (#1829023).

[30] Bernhardt V., Finkelmeier F., Verhoff M.A., Amendt J. (2019). Myiasis in humans—A global case report evaluation and literature analysis. Parasitol. Res..

[31] Nadrah K., Biškup U.G., Špik V.C., Premru M.M., Šoba B. (2021). *Ignatzschineria larvae* bacteremia following *lucilia* sp. Myiasis in an irregular migrant: A case report. Korean J. Parasitol..

